# 3,3′-Diindolylmethane (DIM): A Potential Therapeutic Agent against Cariogenic *Streptococcus mutans* Biofilm

**DOI:** 10.3390/antibiotics12061017

**Published:** 2023-06-06

**Authors:** Yifat Baruch, Karina Golberg, Qun Sun, Karina Yew-Hoong Gin, Robert S. Marks, Ariel Kushmaro

**Affiliations:** 1 Avram and Stella Goldstein-Goren Department of Biotechnology Engineering, Ben Gurion University of the Negev, Beer Sheva 8410501, Israel; yifatbar@post.bgu.ac.il (Y.B.); karingo@bgu.ac.il (K.G.); rsmarks@bgu.ac.il (R.S.M.); 2 Key Laboratory of Bio-Resources and Eco-Environment of the Ministry of Education, College of Life Sciences, Sichuan University, Chengdu 610064, China; qunsun@139.com; 3 Department of Civil and Environmental Engineering, National University of Singapore, Singapore 117576, Singapore; ceeginyh@nus.edu.sg; 4 The Ilse Katz Center for Nanoscale Science and Technology, Ben Gurion University of the Negev, Beer Sheva 8410501, Israel; 5 School of Sustainability and Climate Change, Ben Gurion University of the Negev, Beer Sheva 84105, Israel

**Keywords:** resistance, *S. mutans*, biofilm, caries, 3,3′-Diindolylmethane

## Abstract

Indole, a metabolite of the amino acid tryptophan, has been proven to act as a signal molecule in bacteria, acting in different aspects of biofilm formation. The oral biofilm is a type of biofilm that has consequences for human health. It is a complex, three-dimensional structure that develops on the surface of teeth via the attachment of primary microbial colonizers. Many oral infections are caused by an imbalance occurring in the microorganisms naturally found in oral biofilms and are considered major public health concerns. In this study, we test the effect of a natural bis-indole, 3,3′-Diindolylmethane (DIM), in mitigating the pathogenicity of the oral biofilm inhabiting bacterium *Streptococcus mutans,* a bacterium that is considered to be a principal etiological agent in dental caries. Our study found that DIM was able to attenuate *S. mutans* biofilm formation by 92%. Additionally, treatment with DIM lowered extracellular polymeric substance (EPS) production and decreased its durability significantly under *acidic conditions.* Therefore, the anti-biofilm and anti-virulence properties of DIM against *S. mutans* bacteria in an “oral setting” provides evidence for its usefulness in reducing biofilm formation and potentially for caries attenuation.

## 1. Introduction

Oral microbial biofilms are known to contribute to infectious diseases and are a major public health concern. Oral biofilms are complex three-dimensional structures that develop from a conditioning saliva-derived film, also known as a pellicle, forming on the surface of the teeth. The formation of the pellicle is followed by the attachment of primary microbial colonizers, which are characterized by receptor molecules that are present in the developing pellicle. These early colonizers promote subsequent interactions with secondary colonizers that join the growing biofilm, creating a mature multispecies microbial community [1,2]. One key bacterial component of these biofilms, *Streptococcus mutans,* is considered a principal etiological agent of dental caries [3]. Dental caries is associated with the bacterial metabolism of carbohydrates, leading to prolonged periods of plaque acidification and demineralization of the tooth enamel [4]. Bacterial virulence factors that contribute to dental caries result in stable biofilm formation, acid tolerance, and acid production from carbohydrate metabolism [3].

The major virulence factor for *S. mutans* is its ability to form a biofilm framework that is mainly composed of extracellular polymeric substance (EPS) produced via sugar metabolism [5]. Once *S. mutans* participates in the differentiated community, EPS production promotes a cohesive three-dimensional network. The EPS is a glue-like structure consisting of polysaccharides, extracellular DNA, acids, and proteins [6]. The secretion of EPS by oral bacterial species, including *S. mutans*, mediates bacterial adherence onto the tooth surfaces, thus contributing to the formation of dental plaque biofilms [7]. Another virulence factor includes a membrane-bound F1Fo-ATPase system that pumps protons from cells, while maintaining the internal bacteria’s pH value, resulting in *S. mutans’* acid tolerance [8].

Since biofilm formation by *S. mutans* plays a crucial role in caries promotion, its disruption and removal may be crucial in oral hygiene. Currently, several strategies have been developed against each stage of biofilm formation, including tooth coatings that prevent bacterial attachment and EPS production in addition to mechanical elimination and/or chemical controls [9]. The most common strategy to treat cariogenic biofilms is based on non-specific mechanical brushing and flossing in concert with the use of toothpastes and mouthwashes [10,11] containing antiseptic compounds such as chlorhexidine, fluorides, essential oils, and cetylpyridinium chloride. These treatments result in non-selective oral flora eradication [9,12].

A novel strategy to prevent or diminish dental plaque derived from *S. mutans* biofilms is the use of natural anti-biofilm agents. Indole is known as a metabolite of the amino acid tryptophan produced by many gut and environmental bacteria [13]. Indole is a key player that has been proven to participate in interspecies and interkingdom signaling as well as bacterial pathogenesis [13,14,15]. It affects biofilm formation [16], virulence [17], and the antibiotic resistance of a number of pathogens [18]. A number of indole derivatives, including naturally occurring and synthetic ones, have been reported to act as signaling molecules that affect the behavior of different bacteria [14]. In addition, several indole derivatives have been reported to have more potent antimicrobial and anti-biofilm activities than a basic indole such as 3,3′-Diindolylmethane (DIM) [19,20,21,22]. Furthermore, recent evaluations of a number of natural compounds that affect *S. mutans* biofilm and virulence factors have suggested that these indole compounds may have therapeutic properties [23,24,25,26]. Therefore, the purpose of this study is to evaluate the possibility of harnessing DIM as a therapeutic anti-biofilm and anti-virulent compound against *S. mutans*, allowing evaluation of its potential implementation in mitigating pathogenic biofilm and caries in the oral cavity.

## 2. Materials and Methods

### 2.1. Bacterial Culture and Growth Conditions

*Streptococcus mutans* (ATCC 25175) was cultivated for 24 h in Brain Heart Infusion (BHI) medium (HiMedia) with shaking (140 rpm) at a constant temperature of 37 °C. For acid tolerance assays, a tryptone–yeast extract medium containing 20 mM glucose (TYEG) was applied.

### 2.2. Pre-Coating with Saliva for Biofilm Assay

An amount of 2 mL of whole saliva was collected on ice from a healthy volunteer. Saliva was mixed 1:1 ratio *v*/*v* with AB buffer (50 mM KCl, 1 mM potassium phosphate (0.35 mM K_2_HPO_4_ plus 0.65 mM KH_2_PO_4_), 1 mM CaCl_2_ and 0.1 mM MgCl_2_). An amount of 1 μL of 0.2 M phenylmethyl-sulfonyl-fluoride (PMSF) was added to the mixture and then centrifuged (5500× *g*, 4 °C, 10 min). The supernatant (clarified whole saliva) was collected and filtered through a 0.22 μm PES low protein-binding filter [27]. An amount of 50 μL of clarified saliva was placed in a 96-well for about 18 h at 37 °C. Unbound saliva was removed by blotting the plate on a clean absorbent paper.

### 2.3. Biofilm Growth under Static Conditions

To assess the potential efficacy of DIM against biofilm formation, the *S. mutans* strain was tested under static growth conditions. An overnight culture of *S. mutans* suspensions was diluted 1:100 and incubated for ~3 h until it reached an early exponential phase (OD600~0.2). Subsequently, the culture was further diluted 1:100 and 200 μL of the suspension was placed in a glass bottom 96-well plate, which was previously pre-coated with human saliva as specified above (Thermo Fisher, Waltham, MA, USA). DIM (Sigma-Aldrich, St. Louis, MO, USA) was dissolved in DMSO and supplemented in different concentration ranges (50, 5, 0.5, and 0.05 μM). The cultures were statically incubated at 37 °C for 24 h. For confocal laser scanning microscope (CLSM) visualization, biofilms were stained using a LIVE/DEAD BacLight viability staining kit (Molecular Probes Inc., Eugene, OR, USA) according to the manufacturer’s instructions. As for scanning electron microscope (SEM) imaging, biofilms were allowed to form on a glass slide (1 × 1 cm) in the same condition as mentioned above.

### 2.4. Microscopy and Image Analysis by CSLM

After 24 h, planktonic cells were removed by washing twice with PBS before staining. Biofilms were visualized using a FV1000 CLSM (Olympus, Tokyo, Japan) equipped with a 60 × 1.35 NA lens. Image scanning was carried out using the 488 and 541 nm lasers for the detection of SYTO 9 and PI, respectively. The emission of green-live dye SYTO9 was collected using a 505–525 band pass filter, whereas for the red dead PI stain, the emission was collected using a 560–660 band pass filter. Biofilm 3D images were reconstructed using IMARIS software (Bitplane AG, Zürich, Switzerland) along with quantitative structural calculations by IMARIS Measurement Pro. For each experiment, at least six random positions in each of the three independent cultures were chosen for microscopic analysis.

### 2.5. Dynamic Biofilm Model

In order to attain a greater understanding of DIM efficacy to prevent biofilm formation under shear flow conditions, a continuous culture flow cell was employed. *S. mutans* biofilm was allowed to form in the presence of DIM (0.5 μM) or an equivalent concentration of DMSO as a control on the inner surface of channels of the Ibidi flow cell (µ-slide sizes of 17 × 3.8 × 0.4 mm) for 48 h at 37 °C [19]. To initiate biofilm growth, the cells were inoculated with an overnight culture of *S. mutans* diluted with 0.9% NaCl to an OD_600_ of 0.05. Following 1 h of incubation under static conditions to allow bacterial adhesion, the flow was introduced with a constant flow rate of 3 mL h^−1^ (Masterflex L/S peristaltic pump, Cole-Parmer, Vernon Hills, IL, USA) of 10% BHI supplemented with 1% sucrose (*w*/*v*) for 48 h. The resulting biofilms were macroscopically visualized using CLSM and 3D processed by IMARIS software as stated previously.

### 2.6. Scanning Electron Microscope (SEM) Biofilm Analysis

The morphological properties of the biofilm were analyzed using scanning electron microscopy (SEM Quanta 200, FEI). Bacterial cultures were discarded after 24 h of incubation and the samples were prepared for SEM studies as follows. After fixation in 2.5% buffered glutaraldehyde, the samples were subsequently dehydrated via an ascending, serial ethanol gradient (50, 70, 80, 90, 95, and 100%) and immersed in a hexamethyldisilazane (HMDS)/ethanol gradient solution. The treated and control specimens were air-dried for 4 h, followed by sputter coating with a 20 nm layer of gold using the EMITECH K575× sputtering device (Emitech Ltd., South Petherton, UK). The images were observed under an FEI ESEM Quanta 200 instrument at magnifications ranging from × 2500 to 5000, using an accelerating voltage of 20 kV.

### 2.7. Extracellular Polymeric Substances (EPS) Measurement

For EPS quantification, biofilms of *S. mutans* were produced with different concentrations of DIM in 2 mL BHI, supplemented with 1% sucrose (*w/v*) in 24-well plates. The EPS of biofilms was determined by the “Anthrone method” [24]. Briefly, biofilms were collected by sonication in PBS buffer; then, the precipitate was obtained by centrifugation of 200 μL of the supernatant mixed with 600 μL of “Anthrone reagent” (Sigma-Aldrich) at 95 °C for 5 min. The absorbance at 625 nm was monitored and the concentrations of EPS were calculated using standard curves of dextran. The experiments were repeated three times independently.

### 2.8. Acid Tolerance Assay

The effect of DIM on the acid tolerance of *S. mutans* was evaluated by measuring the viability of the bacteria after 120 min exposure of pH 5.0 [28]. *S. mutans* was grown overnight in TYEG broth and then diluted by 1:20 until mid-logarithmic phase (OD = 0.4–0.5). After centrifugation, the cells were resuspended in TYEG broth buffered with 40 mM phosphate/citrate buffer (pH 5.0) containing 0.5 µM DIM, and incubated at 37 °C for 2 h. The control mixture was DMSO. Samples were removed before and after incubation at pH 5.0 for viable counts. The experiments were repeated three times independently.

## 3. Results and Discussion

Dental caries is one of the most common oral diseases, imposing a large economic burden on many segments of the population worldwide [29]. Dental caries is considered a chronic infectious disease that continues throughout life with high incidence and prevalence. Indeed, up to 90% of the world population has experienced one or more carious lesions during their lifetime [30]. Dental caries is formed through a dysbiosis of the oral microbiota found in the biofilm on the tooth surface [31,32]. This dysbiosis occurs via a shift of the microbial community towards cariogenic bacteria with characteristics of acidogency and acidurity due to high carbohydrate intake [5]. Although *S. mutans* is not the most abundant species in the oral microbiome, it is one of the most prominent cariogenic biofilm producers due to its durability in a high sugar and low pH environment [33]. Therefore, in order to progress towards the eradication of dental caries, alternative therapeutic approaches have been fostered. Recently, we explored the effects of a bis-indole derivative, DIM, on the biofilm formation of different Gram-negative pathogens. This compound was found to be a potential candidate for anti-biofilm properties and virulent attenuation [19]. In general, indole plays important roles as a signal molecule participating in biofilm formation and in multiple physiological pathways in many bacteria [13]. In previous studies, Oh et al. (2012) and Manoharan et al. (2018) showed that indole or indole derivatives attenuate *Candida albicans*, an important member of the flora in the oral cavity [34,35]. Fungal virulence is regulated by indole, mediating filamentation and biofilm formation. Based on these observations, DIM was assessed for its ability to attenuate a single species biofilm of *S. mutans* on glass surfaces.

### 3.1. Effects of DIM on Biofilm Formation of S. mutans

**Figure 1:**
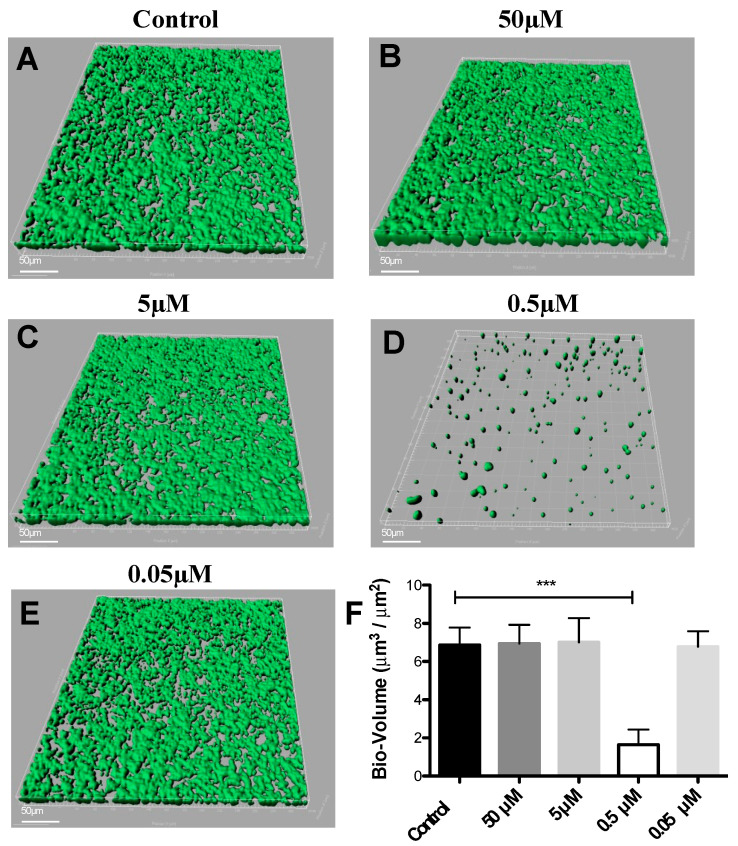
**Dose response of 3,3′-Diindolylmethane on biofilm formation.** CLSM images of biofilm formed by *S. mutans* after 24 h of treatment by different concentrations of (**B**–**E**) DIM or (**A**) untreated (DMSO control). Biofilm formed on a glass bottom 96-well plate after static incubation coated with clarified saliva. CLSM images visualize viable cells stained green and dead cells stained red with the BacLight^®^ LIVE/DEAD Kit, scanned areas of ~318 μm × 318 μm. (**F**) Statistical analysis showing the means ± SDs of biofilm volume generated from three independent sets of experiments (*t*-test; *** *p* < 0.001). Differences were also analyzed for their significance by using one-way ANOVA with Tukey’s test.

**Figure 2:**
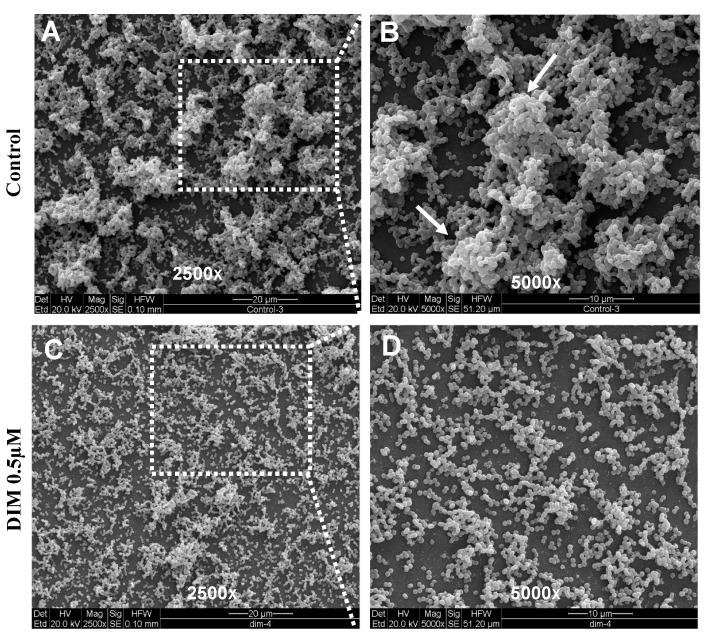
**Surface morphology of biofilm formation**. SEM images of the biofilm formed by *S. mutans* after 24 h (**A**,**B**) untreated (DMSO control) or (**C**,**D**) treatment with 0.5 µM of DIM. Biofilm formed on a glass coated with clarified saliva after static incubation. White arrows indicate the presence of mature microcolonies in the control group. Images are shown at 250× and 5000× magnification.

DIM was tested in a dose-dependent manner (50 μM, 5 μM, 0.5 μM, and 0.05 μM), (Figure 1B–E). Treatment with 0.5 μM DIM under static conditions demonstrated considerably thinner biofilms, being inhibited by 92% (Figure 1D). When compared with the thicker 3D morphology of the control biofilm using SEM imaging (Figure 2A,B), 0.5 µM of DIM also showed a major inhibition of biofilm formation as scattered bacterial growth was observed (Figure 2C,D). This observation was further supported by live bio-volume quantification. In the control, the live bio-volume of *S. mutans* (6.87 μm^3^/μm^2^) was significantly higher than in those under the DIM treatment (1.64 μm^3^/μm^2^) (Figure 1F). Interestingly, neither higher nor lower concentrations showed inhibition, and only 0.5 µM was an effective concentration at retaining apparent anti-biofilm properties against *S. mutans*. Dead bio-volumes in all dosages were not detected, eliminating the possibility of antimicrobial activity elicited by DIM treatment. These findings are in the line with a recent study by Kim et al. that reported the prominent anti-biofilm activity of DIM against acne-causing bacteria, *Cutibacterium acnes* [20]. Out of 20 indole derivatives, both natural and synthetic 7-azaindole, 7-benzyloxyindole, indole-3-carbinol, and DIM significantly inhibited biofilm formation. The latter was found as a highly potential candidate to treat multispecies biofilm-associated infections [20].

**Figure 3:**
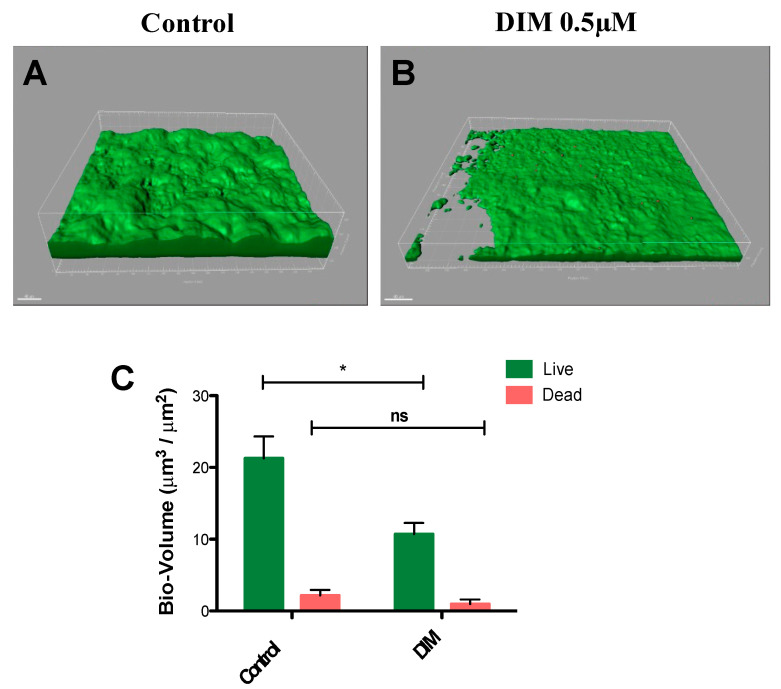
**Biofilm dispersal in dynamic culture**. *S. mutans* biofilm (**A**) control (equivalent concentration of DMSO) or (**B**) 0.5µM DIM continuously supplemented through the inlet media. Biofilms were stained with the LIVE/DEAD bacterial viability kit and subsequently imaged by CSLM 48 h post inoculation. (**C**) Bio-volume (μm^3^/μm^2^) quantification is based on image analysis by IMARIS software. Images were acquired from three different areas for each treatment (means ± SDs), with at least three independent repetitions. Asterisks indicate significant differences compared to control (*t*-test; * *p* < 0.05).

In common caries prevention and management treatments, most of the chemotherapeutic strategies are based on suppressing the levels of cariogenic bacteria by using antimicrobials [36,37]. However, the lack of selectivity found in these therapeutics harms both the pathogenic and natural and beneficial microflora. Moreover, mature differentiated biofilms become recalcitrant and difficult to remove by these antimicrobial agents [37]. Thus, natural molecules such as DIM which are devoid of selection pressure properties provide potential candidates in treating caries. To further this premise, we tested the prominent activity of DIM against biofilms of *S. mutans* under dynamic conditions similar to those found in the oral cavity. Flow cells provide a tool for in vitro cultivation and evaluation of bacterial biofilms under hydrodynamic conditions of flow [38]. The flow cell is designed to enable biofilms to grow in an environment that mimics the hydrodynamic conditions of the oral cavity [39]. Furthermore, since the oral cavity salivary flow (and amount) may affect adherence of bacteria to the substrate [40], the flow cell results are likely to provide a better reflection of what occurs in the mouth. Therefore, we examined the effect of DIM on S. *mutans* biofilm formation in a flow cell model for a period of 48 h (Figure 3A,B) and demonstrated that DIM provided a 50% inhibition of live biofilm bacteria and no significant difference in the dead biofilm bacteria (Figure 3C). Furthermore, dead staining by using propidium iodide did not show an increase in bacterial death as a result of the treatment, suggesting that biofilm inhibition was most likely not due to an antimicrobial effect.

### 3.2. Assessing Anti-Virulence Properties of DIM

**Figure 4:**
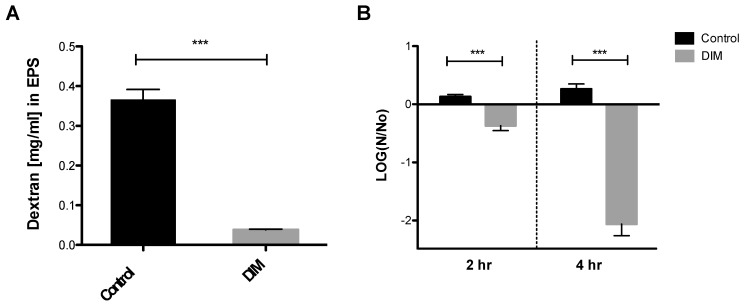
**Quantitative EPS production and aciduricity determination.** (**A**) Biofilm’s EPS of *S. mutans* assessment after 24 h treated with 0.5 µM of DIM or an equivalent amount of DMSO as the control using Anthrone method [24]. Standard curve was prepared with dextran standard with various concentrations (data not shown). (**B**) Acid tolerance was determined by measuring the survival rate of *S. mutans* at pH 5.0. in the presence of 0.5 µM DIM compared with the untreated control (DMSO). N0 and N represent CFU counts before and after 2 h and 4 h treatments in pH 5.0 culture, respectively. Asterisks indicate significant differences compared to control (*t*-test; *** *p* < 0.001).

The oral biofilm bacterial residents are embedded in a self-produced extracellular polymeric matrix (EPS) that provides a fundamental scaffold for cariogenic biofilms, and are recognized as essential virulence factors associated with dental caries [41,42]. Tooth surface colonization takes place once the *S. mutans* cells produce a glue-like extracellular polymer glucan that promotes the buildup of biofilms. The foremost substrate for glucan synthesis, sucrose, has long been regarded as the most cariogenic of all carbohydrates [43]. Our results indicated that 0.5 µM of DIM disrupted the ability of *S. mutans* to synthesize EPS and to form a biofilm (Figure 4A). Indeed, we showed that *S. mutans* treated with DIM markedly reduced the covering of the EPS matrix by 90%. In this context, biofilm attenuation by DIM probably occurred due to a reduction in EPS synthesis that resulted in less adhesion of cells to the surface.

In the oral cavity, sucrose and other carbohydrates in the biofilm matrix are fermented to produce acid, which in turn converts the milieu into a highly acidic microenvironment [43]. The acidic stress increases, thereby creating a favorable niche for other acidogenic and aciduric species to thrive, resulting in the reduction of microbial diversity [44]. Subsequently, the deposition of this acidic biofilm results in the degeneration of the tooth enamel and to dental caries. The mechanism whereby caries is formed is by oral microflora producing lactic acid that causes demineralization of the calcium and phosphate present in the crystal form of hydroxyapatite that comprises the enamel of the teeth [45]. Examination and characterization of the virulence properties expressed by *S. mutans* reveal how this characteristic may actually favor bacterial propagation and explain its ability to thrive in a place that many competing oral bacteria find lethal [46]. *S. mutans’* ability to tolerate this acidic milieu is considered a major virulence factor. Therefore, we tested the *S. mutans* survival rate after 2 and 4 h at pH 5.0 with and without DIM treatment (Figure 4B). Treatment with 0.5 µM DIM decreased *S. mutans* viability significantly after 2 and 4 h, when compared to the control where bacterial viability did not change significantly. Thus, in an acidic environment similar to what is found in the oral cavity, fewer *S. mutans* bacteria survived in the presence of DIM (Figure 4B).

This study demonstrates the possible usefulness of DIM in mitigating pathogenic oral biofilms and consequently chronic dental caries. It is important to note that DIM also has been classified as an anti-cancer agent involved in eradicating various solid malignancies [47] without imposing toxicity to normal cells [48,49]. Therefore, these reports of its low toxicity hold an important advantage in therapeutics as its use in treatment entails minimal complications.

The prevention of the ability of *S. mutans* to produce EPS using this compound may help to change the environment for the development of caries, thus improving oral disease management for the whole population. Additional studies regarding structure and function should be carried out in order to further clarify the possible mechanism of action of DIM. In addition, despite its low toxicity, the possible systemic absorption of DIM should be minimized and a target formulation should be developed for local application.

## Data Availability

Not applicable.
